# TIR-domain-containing protein C as modulator of innate immune checkpoints

**DOI:** 10.1038/s41598-025-29677-w

**Published:** 2025-11-27

**Authors:** Leon Heine, Hannah Griffiths, Huiyun Hu, Isabelle Müller, Svetlana Kuhn, Luca Rudolph, Xaver Rait, Anna Waldhuber, Ying Chen, Thomas Miethke

**Affiliations:** 1 Institute of Medical Microbiology and Hygiene, Medical Faculty Mannheim, Heidelberg University, Theodor-Kutzer-Ufer 1-3, 68167 Mannheim, Germany; 2 Mannheim Institute for Innate Immunoscience (MI3), Franz-Volhard-Str. 6, 68167 Mannheim, Germany; 3 Bavarian Nordic GmbH, Fraunhoferstr. 13, 82152 Planegg, Germany

**Keywords:** *Escherichia coli* strain CFT073, TcpC, Innate immune checkpoint, Toll-like receptor, Cytokine response, Immunology, Microbiology

## Abstract

The TIR-domain-containing protein C (TcpC), a virulence factor of the uropathogenic *Escherichia coli* strain CFT073, binds to crucial components of the toll-like receptor-signaling cascade and the NLRP3 inflammasome thus manipulating these innate immune checkpoints and their immune responses. Here we show that TcpC intensified TLR4-dependent pro-inflammatory cytokine secretion by human epithelial cells upon infection with CFT073. Furthermore, TcpC amplified immune responses of monocytic THP-1 cells and peripheral blood mononuclear cells and monocytes during infection. Interestingly, differentiation of monocytes to M0 macrophages reduced the influence of TcpC on innate immune responses. Similarly, polarization of monocytic THP-1 to M1 macrophages impaired partially the ability of TcpC to modulate cytokine secretion. Induced expression of TcpC revealed that increasing levels of TcpC induction augmented the stimulation of THP-1 cells during infection. In contrast, transfer of TcpC-containing culture supernatants from CFT073 to endotoxin plus ATP-stimulated monocytic THP-1 cells revealed the inhibitory function of TcpC. In summary, we show here that TcpC strengthens cytokine release during infection but dampens it during endotoxin-stimulation.

**Supplementary Information:**

The online version contains supplementary material available at 10.1038/s41598-025-29677-w.

## Introduction

Uropathogenic *Escherichia coli* strains of the phylogenetic group B2 including the strain CFT073 contain the TIR-domain-containing protein C (*tcpC*) in their genome^1,2^. The protein binds to components of two crucial pattern recognition receptors, i.e. Toll-like receptors (TLR) and the NOD-like receptor family pyrin domain containing 3 (NLRP3) inflammasome. In particular, TcpC interacts with TLR4 and MyD88 as well as NLRP3 and caspase-1 modulating secretion of cytokines such as TNFα or IL-1β^2,3^. In addition to its interaction with MyD88, TcpC also lowered MyD88 levels in kidney macrophages isolated from mice with CFT073-induced pyelonephritis and enhanced co-localization of MyD88 with the proteasome marker PSMD2^4^. Further exploration revealed that TcpC acted as an E3 ubiquitin ligase and promoted ubiquitination of MyD88 and its co-localization with the proteasome^4^. This function also impairs extracellular trap formation of neutrophils in CFT073 infected kidneys^5^.

TcpC is an important virulence factor during pyelonephritis caused by *E. coli*. Thus, a previous study analyzed a possible correlation of *E. coli* pathogenicity islands (PAIs) with the severity of urinary tract infections, i.e. asymptomatic bacteriuria, cystitis, pyelonephritis, or urosepsis. The study revealed that only PAI CFT073-*pheU* and PAI CFT073-*serU* associated significantly with urosepsis and pyelonephritis^6^. PAI CFT073-*pheU* and PAI-CFT073-*serU* harbor the virulence genes for P-fimbriae and TcpC, respectively^2,6^. We observed a correlation of the frequency of *E. coli* strains isolated from the urine of patients harboring the *tcpC* gene and the severity of urinary tract infection, i.e. the frequency was highest in patients suffering from pyelonephritis^2^. In mice, urinary tract infections with CFT073 but not with the *tcpC*-deficient CFT073*ΔtcpC* mutant induced kidney abscesses, demonstrating the relevance of TcpC as a virulence factor^2,4,7^. The abscesses were characterized by invading polymorphonuclear neutrophils and bacteria. Interestingly, CFT073- compared to CFT073*ΔtcpC*-infected mice displayed higher numbers of polymorphonuclear neutrophils as well as higher MIP-2 levels in their urine^7^. These findings indicated that TcpC also induced a stronger immune response aside from its negative influence on secretion of proinflammatory cytokines^2,4,7–9^.

Uropathogenic *E. coli* strains of the phylogenetic group B2 are not unique in harboring a TIR-domain-containing protein, as bacteria like *Brucella spp.*^10–12^, *Salmonella enterica* serovar *Enteritidis*^13^, *Yersinia pseudotuberculosis*^14^, *Yersinia pestis*^15^ and *Staphylococcus aureus* MSSA476^16^ contain proteins with similar function^8^.

In addition to its influence on innate immunity, TcpC acts as NAD^+^-consuming enzyme, a function TcpC shares with other bacterial TIR-domain-containing proteins from *Staphylococcus aureus* (TirS), *Acinetobacter baumannii* (AbTir), *Brucella* (BtpA), *Paracoccus dentrificans* (PdTir), *Enterococcus feacalis* (TcpF)^17^ and with the eukaryotic TIR-domain-containing protein sterile alpha and TIR motif containing 1 (SARM1)^18^. TcpC-mediated NAD^+^ depletion could thus harm eukaryotic host cells and bacterial cells metabolically. In addition, the discovery of NAD-capped mRNAs and regulatory small RNAs in *E. coli* provides a mechanism how NAD^+^ shortage may influence the bacterial transcriptome^19^.

These new findings prompted us to re-analyze the influence of TcpC on innate immune responses of cells of the urinary tract. We now report that TcpC from CFT073 stimulates cytokine secretion by the human bladder cell line T24/83, the human monocytic cell line THP-1 and peripheral blood human monocytes. Upon induced expression of TcpC, we provide evidence that stimulation versus inhibition of innate immune responses by TcpC depends on the ratio of bacterial cell numbers and thereby the amount of pathogen-associated molecular patterns and the expression level of TcpC. We also show that transfer of TcpC-containing bacterial culture supernatants impairs cytokine release by endotoxin plus ATP-stimulated THP-1 cells. Thus, TcpC enhances cytokine responses during an infection but dampens these responses during pathogen-associated molecular pattern (PAMP)-stimulation.

## Materials and methods

### Cell culture

THP-1 cells, peripheral blood mononuclear cells and peripheral blood monocytes were cultured in Clicks RPMI (Gibco or Sigma Aldrich) supplemented with 10% FCS and 2 mM L-glutamine. We cultured T24/83 and HK-2 cells in McCoy’s 5 A (modified) (Gibco or Sigma Aldrich) or RPMI-1640 medium (Gibco) supplemented with 10% FCS. Adherent cell lines were washed with PBS (Sigma Aldrich), dissociated from the cell culture flask with a Trypsin-EDTA solution (Sigma Aldrich), 10-fold diluted with PBS, resuspended in fresh medium and seeded into new flasks twice or thrice a week. We cultured all cell lines at 37 °C and 5% CO_2_.

### Differentiation of THP-1 cells

We differentiated THP-1 cells from monocytes to M0 macrophages using PMA (Phorbol-12-myristat-13-acetat). Depending on the experiment, the cells were seeded with different PMA concentrations (between 0.0005 and 200 ng/ml) and incubated for 24 h to 72 h before they were washed thrice with PBS and left to rest for 24 h in PMA-free culture medium.

Polarization of THP-1 cells to M1 or M2 macrophages was performed as previously described^20^. In short, cells were differentiated into M0 macrophages using 100 ng/ml PMA for 24 h, subsequently washed thrice with PBS and rested for 24 h in PMA free culture medium. To obtain M1 macrophages we polarized M0 macrophages with 20 ng/ml IFN-γ and 10 pg/ml LPS for 24 h. We used 20 ng/ml IL-4 and 20 ng/ml IL-13 for 48 h to polarize the cells to M2 macrophages. After polarization, we washed the cells thrice with PBS and rested them 24 h in PMA-free culture medium. Infections were done directly after the resting time.

### PBMC isolation from Buffy coats

Buffy coats from healthy donors were diluted 1:3 with PBS and PBMCs were isolated via density gradient centrifugation using Biocoll (Bio&Sell, #BS.L6715). Centrifugation was performed at 400 x g for 30 min at room temperature. After centrifugation, cells were washed three times with PBS. The use of buffy coats was ethically approved by the ethic commission II of the Medical Faculty Mannheim, Heidelberg University, file 2025 − 649.

### Monocyte isolation

Primary human monocytes were isolated from PBMCs by CD14 positive selection using CD14 microbeads (Miltenyi Biotech, #130-050-201; MS Columns, Miltenyi Biotech, #130-042-201) according to the manufacturer’s instructions. The purity of positively selected monocytes was confirmed via FACS analysis using CD45 (Miltenyi Biotech, #130-110-770), CD11b (Miltenyi Biotech, #130-110-616) and CD282 (Miltenyi Biotech, #130-123-588) antibodies^21^.

### MDM generation

Monocyte-derived macrophages (MDMs) were generated by differentiation of monocytes in complete culture medium (RPMI + 10% FCS) supplemented with 10 ng/ml M-CFS (Miltenyi Biotech, #130-096-491) and 1 ng/ml GM-CSF (Miltenyi Biotech, #130-093-862) for 6 days. The medium was changed every 2–3 days.

### Isolation of murine bone marrow derived dendritic cells

We prepared conventional BMDCs from femora and tibiae of mice. We plated the cells on bacterial petri dishes overnight in culture medium (RPMI 1640, 10% heat-inactivated FCS, 100 IU/ml penicillin, 100 mg/ml streptomycin (PAA Laboratories), and 50 mM 2-ME (Invitrogen) in the presence of 20 ng/ml granulocyte-macrophage CSF (GM-CSF) (Peprotech) at a density of 6 × 10^6^ cells/dish and cultivated them for another 6 days in complete medium in the presence of 20 ng/ml GM-CSF, which was added a second time on day 3.

Bone marrow-derived cells were prepared with permission of the Regierung von Oberbayern, Munich, Germany (file number 55.2-1-54-2532-111-09).

### Propagation of bacterial pathogens

The *Escherichia coli* strains CFT073, CFT073*ΔtcpC*, CFT073*ΔtcpC* + pTcpC and CFT073*ΔtcpC* + pASK-IBA5plus-TcpC were grown overnight in lysogeny broth (LB, 37 °C, shaking 200 rpm). Overnight cultures were washed three times in Clicks RPMI or McCoy’s 5 A medium containing 3% FCS and then used to infect THP-1 cells, peripheral blood mononuclear cells, peripheral blood monocytes, wild type, TLR4- or MyD88-deficient T24/83 cells and HK-2 cells at different multiplicities of infection (MOI).

### Production of bacterial culture supernatants

Overnight cultures of CFT073, CFT073*ΔtcpC* and CFT073*ΔtcpC* + pASK-IBA5plus-TcpC were diluted 1:100 in 20 ml LB medium and cultured until reaching an OD_600_ of 0.5. Afterwards, bacteria were washed with infection medium and either induced with specified amounts of anhydrotetracycline (Atc) or not induced and cultured for 3 h at 37 °C, 200 rpm. Bacteria were then centrifuged at 8000 x g for 5 min and culture supernatants filtered through a 0.2 μm syringe filter (Thermo Scientific, #723–2520). The medium of the supernatants was washed with fresh medium by using an Amicon 10 kDa mwco filter (Merck, #UFC901008). During this process supernatants were concentrated 1:20, however, for treatment of cells they were diluted 1:40 again.

### Western blot

Bacterial cells were incubated with lysozyme (1 mg/ml) at 27 °C for 15 min followed by incubation with equal volume 2x lysis buffer (200mM Tris-HCl pH8, 2% SDS, 20% Glycerol) at 98 °C for 15 min. Soluble proteins were obtained after centrifugation at 16,000 x g for 15 min. After Bradford measurement, equal amounts of proteins were mixed with 4x Laemmli buffer (250 mM Tris-HCl pH6.8, 40% glycerol, 8% SDS, 0.02% Bromophenol Blue, β-mercaptoethanol) and boiled at 95 °C for 5 min. The samples were then separated in 12% SDS-PAGE, and transferred to a nitrocellulose membrane. After blocking in 5% milk-TBST, the blot was incubated with primary antibody (listed below) overnight at 4 °C. After incubation with HRP-conjugated anti-mouse secondary antibody (DAKO, #P0447) or anti-mouse secondary antibody (DAKO, #P0399) at room temperature for 1 h, the blots were covered with 1 ml of WesternBright chemiluminescence substrate Quantum (Biozym, #541015) for 1 min and exposed with an ECL ChemoCam Imager HR6.0 (Intas Science Imaging). The primary antibodies used for Western blots were anti-TcpC rabbit serum raised against the last 35 AS (SKYSHYLADKMALQTSLYSVKEIARELAEIAYRRR) of TcpC (Davids Biotechnology) and anti-GAPDH (Thermo Fisher Scientific, #MA5-15738).

### ELISAs

ELISA DUOset kits against human IL-1β, IL-6, IL-8 and TNFα were purchased from RnD systems (#DY201, #DY206, #DY210, respectively, and ancillary reagent kit #DY008B) and used according to the manufacturer´s protocol. Absorbance was detected and quantified using a TECAN Spark 10 M plate reader and the software Magellan.

### Generation of TLR4- or MyD88-deficient T24/83 cells

The generation ofTLR4- or MyD88-deficient T24/83 cells was done using CRISPR/Cas9. Briefly, two different gRNAs specific for exon 2 and two for exon 3 of TLR4 were cloned into the plasmid p3511 (Addgene plasmid #48138, kind gift from Dr. Georg Stoecklin, Division of Biochemistry, Med. Faculty Mannheim, Germany) using BbsI as restriction enzyme^22^. We used primers gRNA_TLR4_2a_fw, gRNA_TLR4_2a_rv, gRNA_TLR4_2b_fw, gRNA_TLR4_2b_rev, gRNA_TLR4_3a_fw, gRNA_TLR4_3a_rv, gRNA_TLR4_3b_fw, gRNA_TLR4_3b_rv to generate TLR4 exon 2 and TLR4 exon 3 -specific gRNAs (Table 1). The resulting four different plasmids (p3511 + gRNA_TLR4_2a, p3511 + gRNA_TLR4_2b, p3511 + gRNA_TLR4_3a, p3511 + gRNA_TLR4_3b) were verified by restriction digestion with BbsI and EcoRV, and PCR using the primers P3511_sgRNA_seq_fwd and P3511_sgRNA_seq_rev (Table 2) and sequencing for the correct integration of gRNAs. In case of MyD88, primers gRNA_MyD88_up_for, gRNA_MyD88_up_rev, gRNA_MyD88_NEU1_for and gRNA_MyD88_NEU1_rev were used to generate two different gRNAs, which we cloned into the plasmid p3511 + gRNA_MyD88_up and p3511 + gRNA_MyD88_NEU1. Both plasmids were verified by restriction digestion with BbsI and EcoRV, and sequencing for the correct integration of gRNAs using the primers P3511_sgRNA_seq_fwd or P3511_sgRNA_seq_rev (Table 2).

**Table 1:** Sequences of primers for specific guide rnas’ insertion in p3511.

gRNAs	Sequence	Target
gRNA_TLR4_2a_fw	5’-caccGTCCAGGTTCTTGGTTGAGA-3’	TLR4 exon 2
gRNA_TLR4_2a_rv	5’-aaacTCTCAACCAAGAACCTGGAC-3’	TLR4 exon 2
gRNA_TLR4_2b_fw	5’-caccGATAAATCCAGCACCTGCAGTTCT-3’	TLR4 exon 2
gRNA_TLR4_2b_rev	5’-aaacAGAACTGCAGGTGCTGGATTTATC-3’	TLR4 exon 2
gRNA_TLR4_3a_fw	5’-caccGTCTAAAGAGAGATTGAGTA-3’	TLR4 exon 3
gRNA_TLR4_3a_rv	5’-aaacTACTCAATCTCTCTTTAGAC-3’	TLR4 exon 3
gRNA_TLR4_3b_fw	5’-caccGAAGTCCATCGTTTGGTTCT-3’	TLR4 exon 3
gRNA_TLR4_3b_rv	5’-aaacAGAACCAAACGATGGACTTC-3’	TLR4 exon 3
gRNA_MyD88_up_for	5’-caccGGCCGACTGGACCGCGCTGG-3’	MyD88 exon 1
gRNA_MyD88_up_rev	5’-aaacCCAGCGCGGTCCAGTCGGCC-3’	MyD88 exon 1
gRNA_MyD88_NEU1_for	5’-aaacTGGCGCCTCTGTAGGCCGAC-3’	MyD88 exon 1
gRNA_MyD88_NEU1_rev	5’-caccGTCGGCCTACAGAGGCGCCA-3’	MyD88 exon 1

Lower case letters indicate overhangs compatible with BbsI cutting sites.

**Table 2:** Sequences of primers.

Primer	Sequence
P3511_sgRNA_seq_fwd	5’-CTTGGGTAGTTTGCAGT-3’
P3511_sgRNA_seq_rev	5’-GAGCCATTTGTCTGCAG-3’
tlr4_exon2_fw	5’-CCATCTCTGGTCTAGGAGAGG-3’
tlr4_exon2_rev	5’-CAGCCAACTGCCTACTTCACAG-3’
tlr4_exon3_fw	5’-GACCAATCTAGAGCACTTGGAC-3’
tlr4_exon3_rev	5’-CAAGGCTTGGTAGATCAACTTCTG-3’
myd88_exon1_for	5’-CGCCTCGAGACCTCAAGGG-3’
myd88_exon1_rev	5’-GGGACCCGCATGGTTCTCC-3’

In addition to the gRNAs, the plasmid p3511 encodes for Cas9 and GFP. Four plasmids in case of TLR4 and two plasmids in case of MyD88 were transfected into T24/83 cells using Lipofectamin 2000 and GFP-expressing T24/83 cells were enriched two days after transfection via cell sorting. Single clones were obtained by limiting dilution cloning using 96-well plates and 10% (v/v) T24/83 cell-conditioned medium. Growing cells were subsequently tested by PCR using the primers tlr4_exon2_fw, tlr4_exon2_rev, tlr4_exon3_fwd, tlr4_exon3_rev, myd88_exon1_for, myd88_exon1_rev (Table 2) and sequencing of the PCR-products whether mutations were introduced into exon 2 and exon 3 of TLR4 and exon 1 of MyD88.

### Statistics

All statistical analyses were performed using GraphPad Prism 8.4.3 (GraphPad Software, LLC). Statistical comparisons of two groups were analyzed by unpaired t test, of more than two groups by one-way or two-way ANOVA, post hoc test Tukey. *P* values < 0.05 were considered as statistically significant.

## Results

### Stimulatory effect of TcpC

**Fig. 1:**
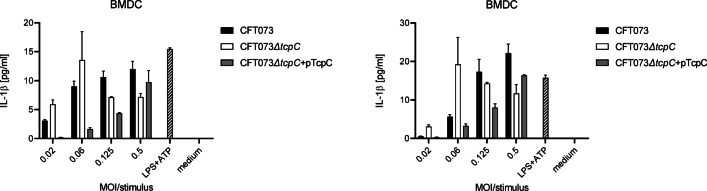
Inhibitory versus stimulatory influence of TcpC in a MOI-dependent manner. We infected BMDC for 3.5 h with CFT073, CFT073*ΔtcpC* or CFT073*ΔtcpC* + pTcpC with different MOIs as indicated and determined IL-1β in the culture supernatant. LPS + ATP served as positive, medium as negative control. Left and right graph show two independent experiments, each bar represents two replicates.

**Fig. 2:**
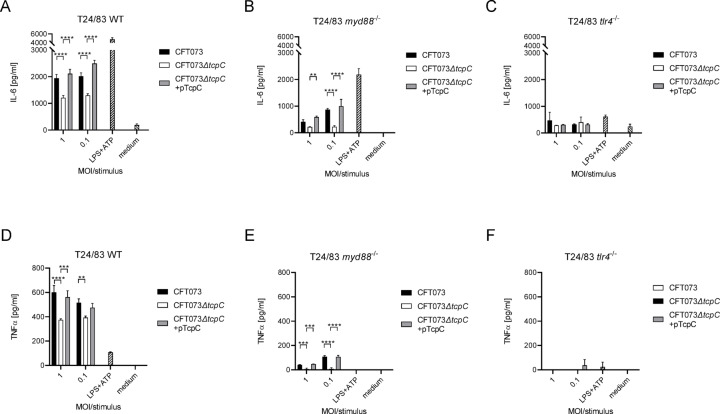
TcpC enforces CFT073 induced TLR4- and MyD88-dependent cytokine secretion by the human bladder epithelial cell line T24/83. Wild type (**A**,**D**), *myd88*^−/−^ (**B**,**E**), and *tlr4*^−/−^ human bladder epithelial T24/83 cells (**C**,**F**) were infected for 5 h with CFT073, CFT073*ΔtcpC* or CFT073*ΔtcpC* + pTcpC with different MOIs as indicated. IL-6 (**A**–**C**) and TNFα levels (**D**–**F**) were determined in the culture supernatant. LPS + ATP served as positive, medium as negative control. The experiments were repeated twice with similar results, each bar represents three replicates. **P < 0.01, ***P < 0.001, ****P < 0.0001, two-way ANOVA, post hoc Tukey.

We reported earlier that TcpC impaired TNFα and IL-1β secretion by RAW264.7 macrophages, bone marrow-derived macrophages (BMDM) and bone marrow-derived dendritic cells (BMDC)^2,3^. However, we also observed at MOIs of 0.5 of CFT073 that TcpC stimulated secretion of proinflammatory cytokines by BMDC (Fig. 1). To further explore the influence of TcpC on innate immune responses, we infected human cells, relevant during a bladder infection, such as the human bladder epithelial cell line T24/83, with CFT073, the *tcpC*-deficient mutant CFT073*ΔtcpC* or the complemented *tcpC*-deficient mutant CFT073*ΔtcpC* + pTcpC. In contrast to our expectation, we found that infection of T24/83 cells with the *tcpC*-deficient CFT073*ΔtcpC* strain resulted in statistically significant lower amounts of IL-6 and TNFα (Fig. 2A, D). We generated *myd88*- or *tlr4*-deficient T24/83 cells to explore whether the infection triggers cytokine secretion via this signaling cascade. *Myd88*-deficiency attenuated secretion of IL-6 and TNFα upon infection with CFT073 (Fig. 2B, E), while *tlr4*-deficiency almost completely abolished secretion of both cytokines (Fig. 2C, F). Interestingly, the stimulatory influence of TcpC was still significant in *myd88*-deficient T24/83 cells (Fig. 2B, E). Taken together, CFT073 stimulated T24/83 cells via TLR4 and MyD88 and TcpC augmented the cellular response.

**Fig. 3:**
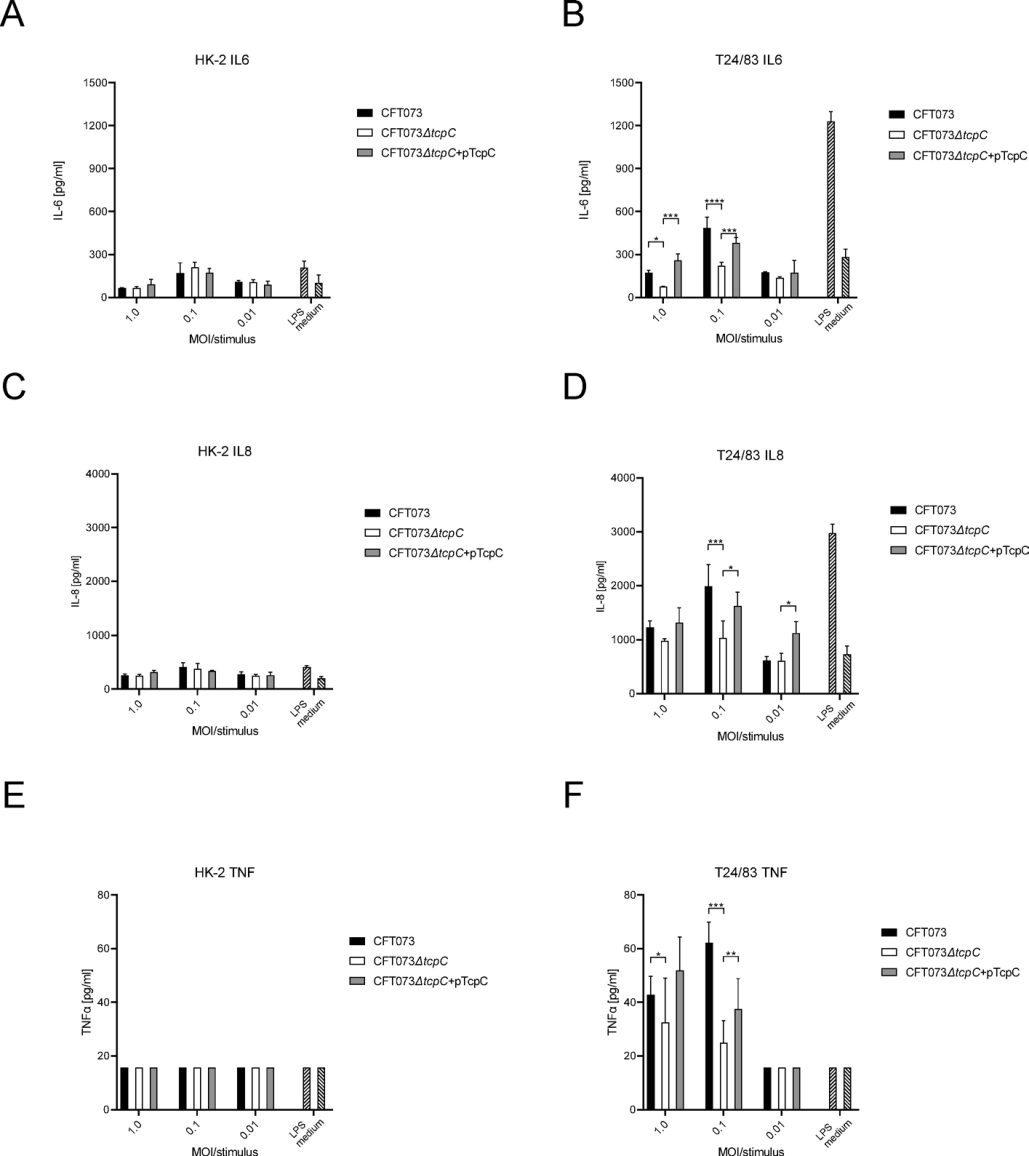
Poor response of HK-2 cells to CFT073 infection. Kidney proximal tube epithelial HK-2 (**A**,**C**,**E**) and human bladder epithelial T24/83 cells (**B**,**D**,**F**) were infected for 5 h with CFT073, CFT073*ΔtcpC* or CFT073*ΔtcpC* + pTcpC with different MOIs as indicated. IL-6 (**A**,**B**), IL-8 (**C**,**D**) and TNFα levels (**E**,**F**) were determined in the culture supernatant. LPS + ATP served as positive, medium as negative control. The experiments were repeated thrice with similar results, each bar represents three replicates. *P < 0.01, ***P < 0.001, ****P < 0.0001, two-way ANOVA, post hoc Tukey.

We then compared the cytokine response of the kidney proximal tube epithelial cell line HK-2 with the response of T24/83 cells to an infection with CFT073 and its modulation by TcpC. Figure 3 shows that HK-2 cells in comparison to T24/83 cells generated a very weak IL-6 and IL-8 response, which was not modulated by TcpC (Fig. 3A–D). In contrast to T24/83 cells, TK-2 cells failed completely to secrete TNFα upon infection with CFT073 (Fig. 3E, F).

### Differentiation of monocytes to M0 macrophages attenuates the influence of TcpC on cytokine secretion

**Fig. 4:**
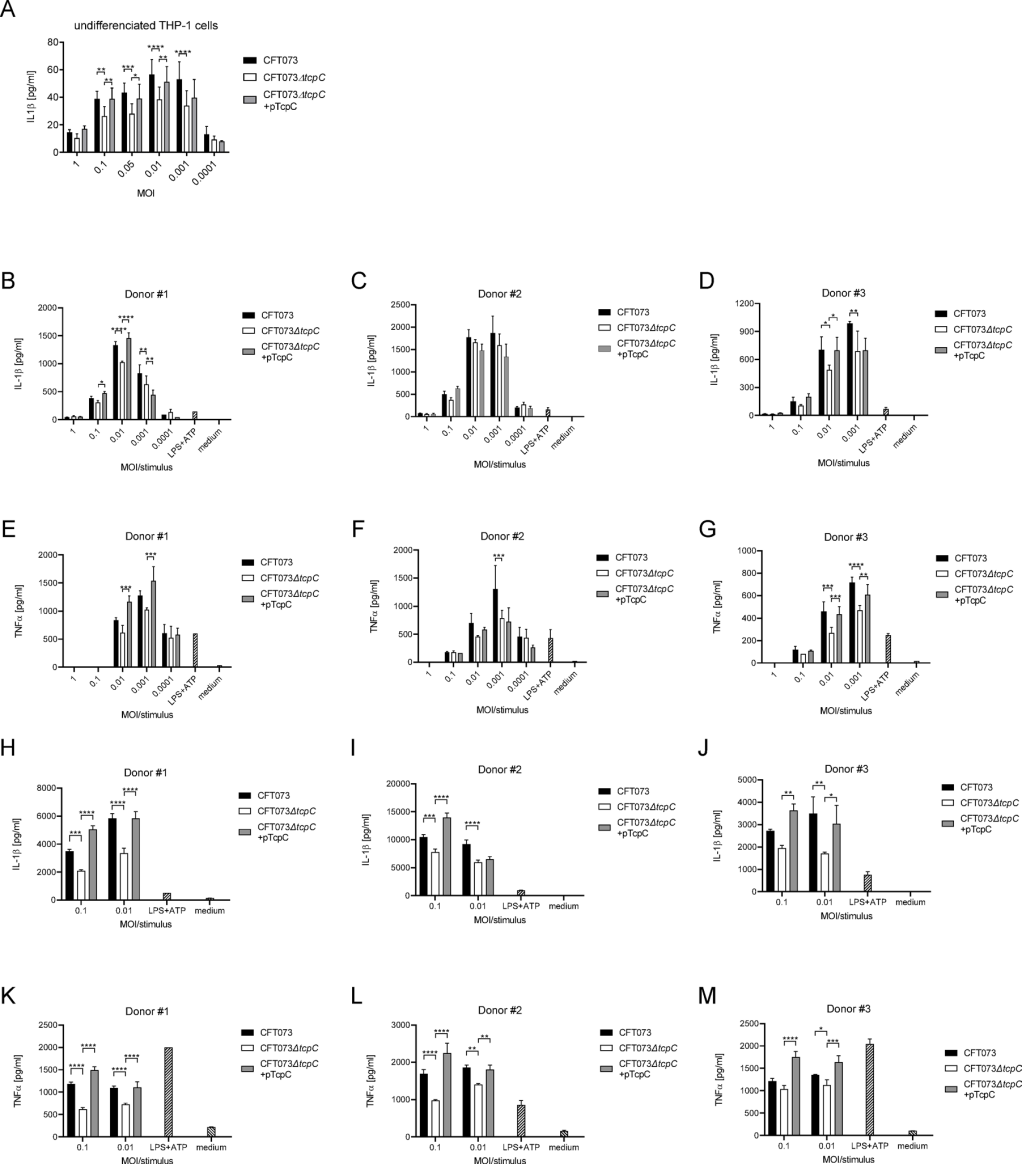
TcpC harnesses CFT073 induced immune responses by monocytic THP-1 cells, PBMC and peripheral blood monocytes. We infected monocytic THP-1 cells for 5 h with CFT073, CFT073*ΔtcpC* or CFT073*ΔtcpC* + pTcpC with different MOIs as indicated and determined IL-1β in the culture supernatant (**A**). The graph in (**A**) depicts data from three independent experiments, each experiment was performed with three replicates. Peripheral blood mononuclear cells from three healthy blood donors were infected for 5 h with CFT073, CFT073*ΔtcpC* or CFT073*ΔtcpC* + pTcpC with different MOIs as indicated. We determined IL-1β (**B**–**D**) and TNFα (**E**–**G**) in the culture supernatant. Graphs in (**B**–**G**) depict three replicates. Peripheral blood monocytes from three healthy blood donors were infected as described for PBMCs and IL-1β (**H**–**J**) and TNFα levels (**K**–**M**) of culture supernatants were determined. Graphs in (**H**–**M**) depict three replicates. LPS + ATP served as positive, medium as negative control. *P < 0.05, **P < 0.01, ***P < 0.001, ****P < 0.0001, two-way ANOVA, post hoc Tukey.

**Fig. 5:**
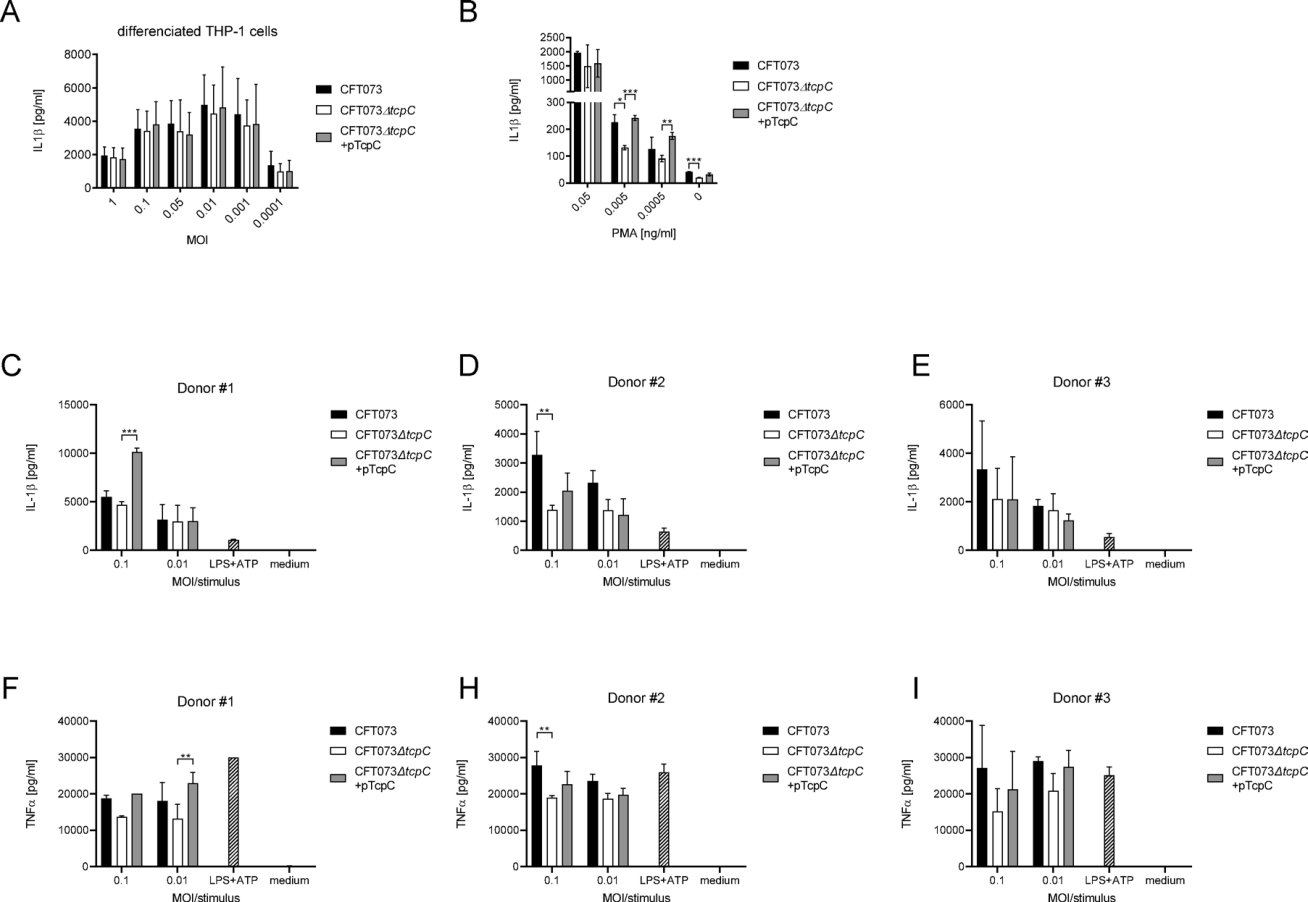
Differentiation of THP-1 cells and peripheral blood monocytes to macrophages reduces the influence of TcpC on immune responses. We infected PMA-differentiated THP-1 M0 macrophages for 5 h with CFT073, CFT073*ΔtcpC* or CFT073*ΔtcpC* + pTcpC with different MOIs as indicated and determined IL-1β in the culture supernatant (**A**). The graph in (**A**) depicts data from three independent experiments, each experiment was performed with three replicates. In (**B**) we titrated the amount of PMA used for differentiation of THP-1 cells, infected the cells for 5 h with CFT073, CFT073*ΔtcpC* or CFT073*ΔtcpC* + pTcpC with a MOI of 0.1 and determined IL-1β in the culture supernatant. The graph in (**B**) depicts three replicates. We differentiated the peripheral blood monocytes to macrophages from the same three healthy blood donors described in (Fig. 4) and infected them as described for THP-1 macrophages. IL-1β (**C**–**E**) and TNFα levels (**F**–**I**) of culture supernatants were determined. The graphs in (**C**–**I**) depict three replicates. LPS + ATP served as positive, medium as negative control. *P < 0.05, **P < 0.01, ***P < 0.001, two-way ANOVA, post hoc Tukey.

Innate immune cells such as tissue resident macrophages are crucial to control bacterial burden during a bladder or kidney infection^23–25^. We therefore explored the influence of TcpC on monocytes and M0 macrophages during an infection with CFT073 in vitro. We found that TcpC augmented IL-1β secretion of the human monocytic cell line THP-1 across a wide range of MOIs (Fig. 4A). We tried to verify this result with peripheral human blood mononuclear cells (PBMC). These experiments revealed that TcpC again increased the CFT073-induced levels of IL-1β (Fig. 4B–D, Fig. S1A-C) and TNFα (Fig. 4E-G, Fig. S1D-F) significantly, however in a donor-dependent manner. Thus, PBMCs of donor #2 are only weakly influenced by TcpC. The reason for this is presently unclear. We then isolated peripheral CD14^+^ blood monocytes and observed a significant TcpC-intensified stimulation of IL-1β (Fig. 4H–J, Fig. S1G-I) in five of six and TNFα (Fig. 4K–M, Fig. S1J-L) in all six donors analyzed. When we differentiated THP-1 cells to M0 macrophages using Phorbol 12-myristate 13-acetate (PMA) we failed to observe a TcpC-mediated further increase of IL-1β secretion during an infection with CFT073 (Fig. 5A). Note that the immune response to the infection was around 100-fold stronger compared to monocytic THP-1 cells (Figs. 4A and 5A). A titration of PMA demonstrated that a concentration of 0.05 ng/ml of PMA sufficed to impair the influence of TcpC on IL-1β secretion by differentiated M0 THP-1 cells (Fig. 5B). Differentiation of the CD14^+^ peripheral blood monocytes to macrophages using M-CSF and GM-CSF reduced the influence of TcpC on IL-1β- (Fig. 5C–E) and TNFα-secretion (Fig. 5F–I) during an infection with CFT073. However, there was still a donor-dependent visible trend. Again, the differentiated versus non-differentiated cells secreted 10-fold higher TNFα amounts (Figs. 4K–M and 5F–I).

### Polarization of THP-1 cells to M1 or M2 macrophages

**Fig. 6:**
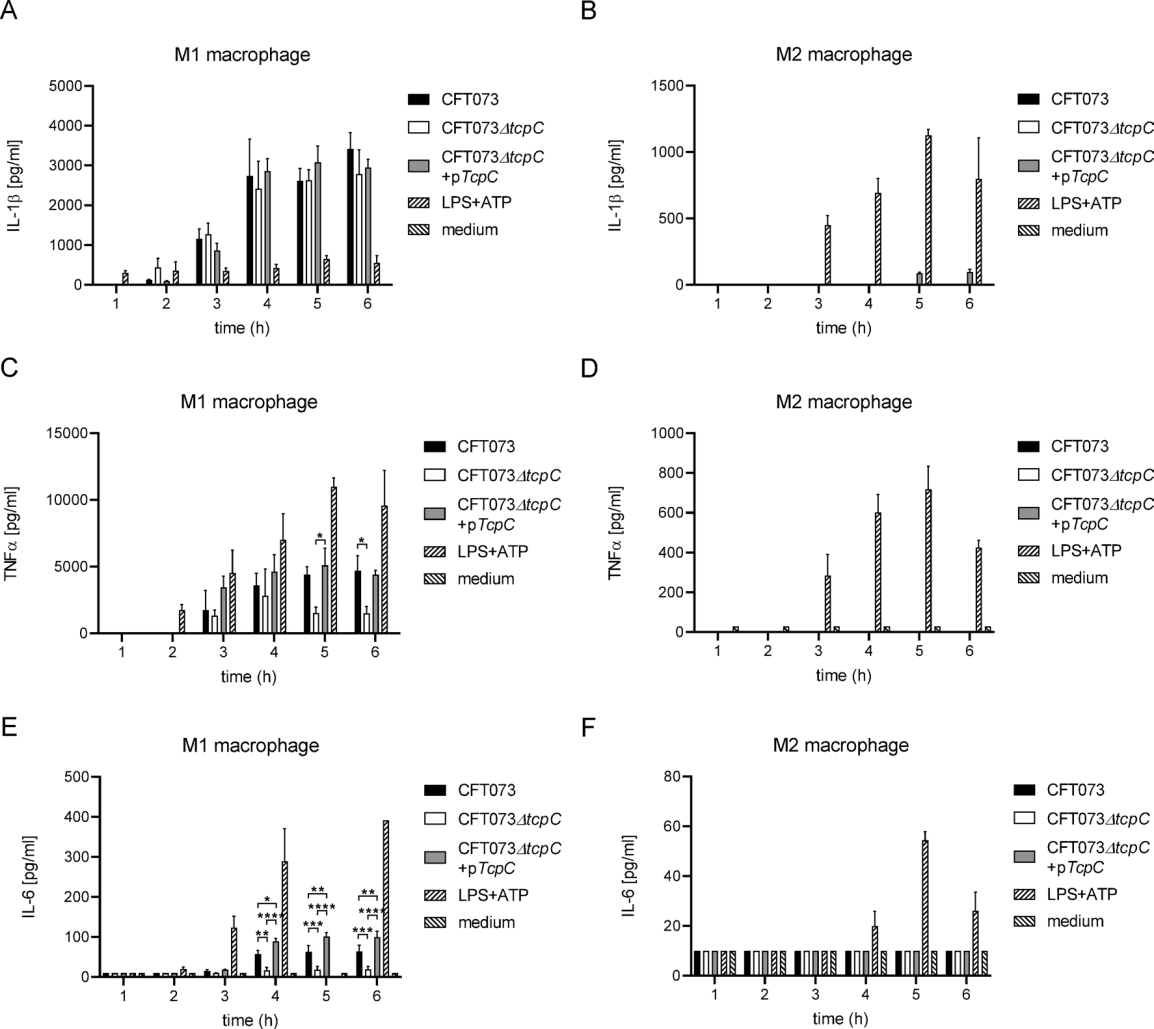
Polarization of THP-1 cells to M1 macrophages prevents the influence of TcpC on IL-1β secretion. We infected PMA/endotoxin/IFNγ/LPS-polarized THP-1 M1 (**Α**,**C**,**E**) and PMA/IL-4/IL-13-polarized THP-1 M2 macrophages (**B**,**D**,**F**) with CFT073, CFT073*ΔtcpC* or CFT073*ΔtcpC* + pTcpC with a MOI of 0.1 for different periods of time as indicated. We determined IL-1β (**A**,**B**), TNFα (**C**,**D**) and IL-6 (**E**,**F**) in the culture supernatant. The graphs in (**A**–**F**) depict two or three replicates. LPS + ATP served as positive, medium as negative control. *P < 0.05, **P < 0.01, ***P < 0.001, ****P < 0.0001, one-way ANOVA, post hoc Tukey (**A**,**D**), post hoc Tukey (**B**).

To analyze further functional states of THP-1 cells, which might influence the sensitivity for TcpC during an infection with CFT073, we polarized THP-1 cells to M1 and M2 macrophages using PMA/IFNγ/LPS or PMA/IL-4/IL-13, respectively. Figure 6A demonstrates that the M1-polarized THP-1 cells responded within 2 h to the infection with IL-1β secretion. TcpC appeared to impair this response, but later no influence was visible. In contrast, the virulence factor augmented TNFα and IL-6 responses (Fig. 6C, E). Interestingly, M2-polarized THP-1 cells secreted these cytokines not at all upon infection with CFT073 (Fig. 6B, D, F). Nevertheless, endotoxin induced their secretion, albeit TNFα and IL-6 levels were considerably lower compared to M1-polarized THP-1 cells (Fig. 6). These findings also demonstrate that the stimulatory potential of CFT073 and *E. coli*-derived endotoxin are obviously not identical.

### TcpC-augmented innate immune stimulation depends on the ratio of bacterial replication and TcpC expression

**Fig. 7:**
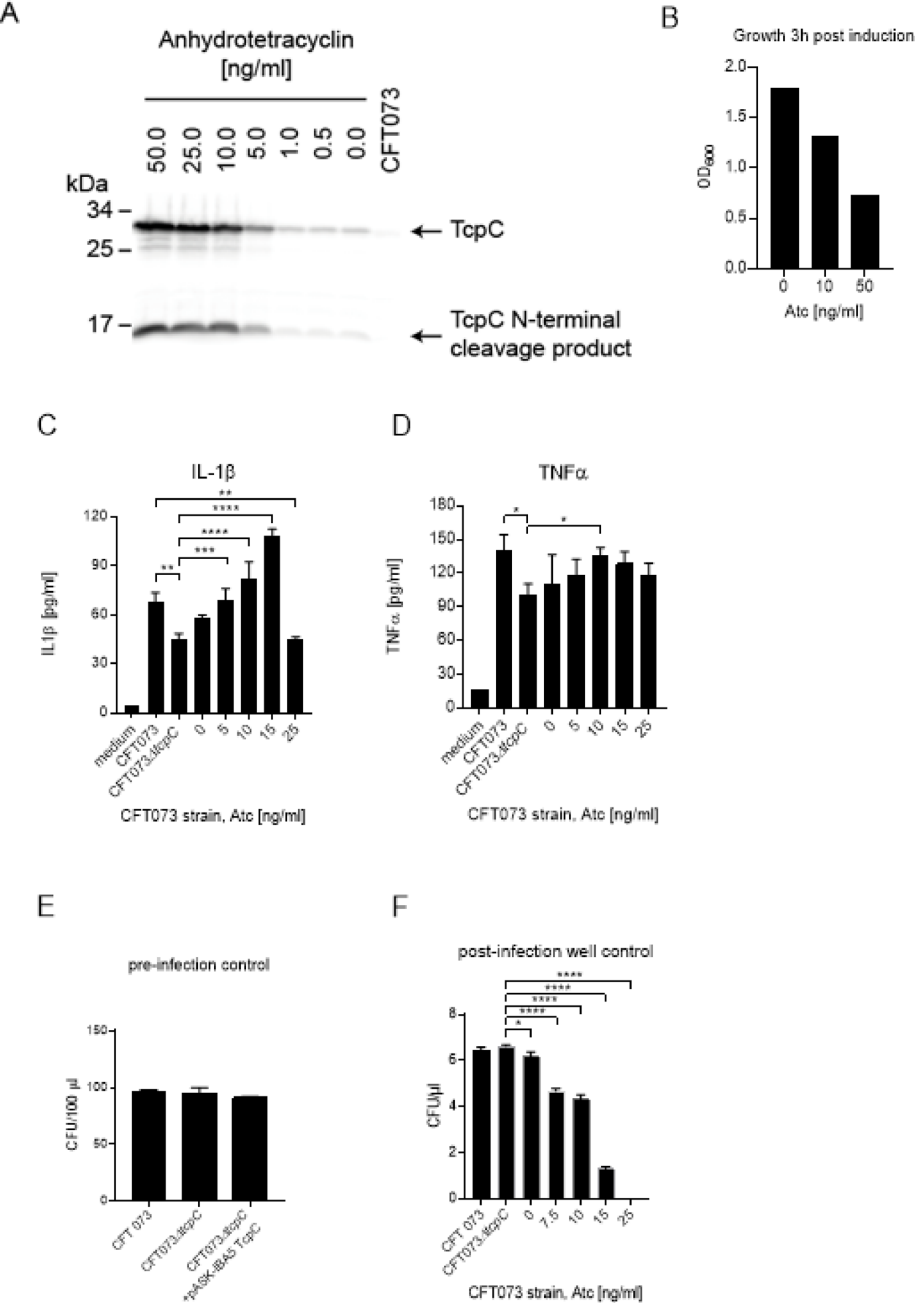
TcpC impairs bacterial replication nevertheless stimulates monocytic THP-1 cells. We induced CFT073*ΔtcpC* + pASK-IBA5-TcpC with titrated doses of Atc and analyzed the cytosolic expression of TcpC by Western blot. We also used CFT073 to explore endogenous expression levels of TcpC. The observed size difference of TcpC expressed by CFT073 versus CFT073*ΔtcpC* + pASK-IBA5-TcpC is due to an inserted Strep-tag in the latter case (**A**). The effect of TcpC induction on bacterial replication is depicted in (**B**), each bar represents one culture. We infected monocytic THP-1 cells with CFT073, CFT073*ΔtcpC*, or CFT073*ΔtcpC* + pASK-IBA5-TcpC (MOI = 0.1), whose TcpC expression was induced with titrated amounts of Atc. We determined IL-1β (**C**) and TNFα (**D**) in culture supernatants. The experiment depicted in (**C**) and (**D**) was performed with three replicates and repeated twice with similar results. We monitored bacterial replication bevor (**E**) and after infection of THP-1 cells (**F**). Graphs in (**E**) and (**F**) depict three replicates. *P < 0.05, **P < 0.01, ***P < 0.001, ****P < 0.0001, one-way ANOVA, post hoc Tukey.

To get further insight into TcpC-augmented innate immune stimulation, we generated the anhydrotetracylin (Atc)-inducible *tcpC* expression plasmid pASK-IBA5plus-TcpC. Transfection of pASK-IBA5plus-TcpC into CFT073*ΔtcpC* and its induction with titrated Atc amounts resulted in a dose-dependent cytosolic TcpC expression in the CFT073*ΔtcpC* + pASK-IBA5plus-TcpC strain (Fig. 7A). Note, that the plasmid is leaky and TcpC levels produced by the uninduced CFT073*ΔtcpC* + pASK-IBA5plus-TcpC strain are higher compared to the level generated by CFT073, which is just visible. Induced expression of TcpC also reduced the replication of the bacterial strain (Fig. 7B). Infection of monocytic THP-1 cells with CFT073*ΔtcpC* + pASK-IBA5plus-TcpC and titrated induction of the plasmid demonstrated that the expression level of TcpC achieved statistically significant higher IL-1β amounts with 5 to 15 ng/ml of Atc compared to CFT073*ΔtcpC* (Fig. 7C), while TNFα amounts were slightly but significantly increased (Fig. 7D). The stimulatory activity of TcpC on IL-1β and TNFα secretion is remarkable, since bacterial numbers, which were identical between the strains at the beginning of the infection (Fig. 7E), were reduced Atc-dose dependently at the end of the culture period of 5 h due to induced TcpC expression (Fig. 7F). The inhibition of CFT073 replication by induced expression of TcpC depended mainly on its TIR-domain as the effect of the N-terminal half of the protein was much weaker (Fig. S2). A higher expression achieved by an Atc concentration of 25 ng/ml reduced IL-1β secretion significantly (Fig. 7C), presumably because of the stronger reduction of the infectious dose during the culture period (Fig. 7F). Taken together, it appears that during an infection with CFT073*ΔtcpC* + pASK-IBA5plus-TcpC strain the ratio between the amount of TcpC and the number of bacteria present during the culture period is crucial for maximal stimulation of IL-1β secretion.

### Effect of TcpC on endotoxin plus ATP-driven innate immune responses

**Fig. 8:**
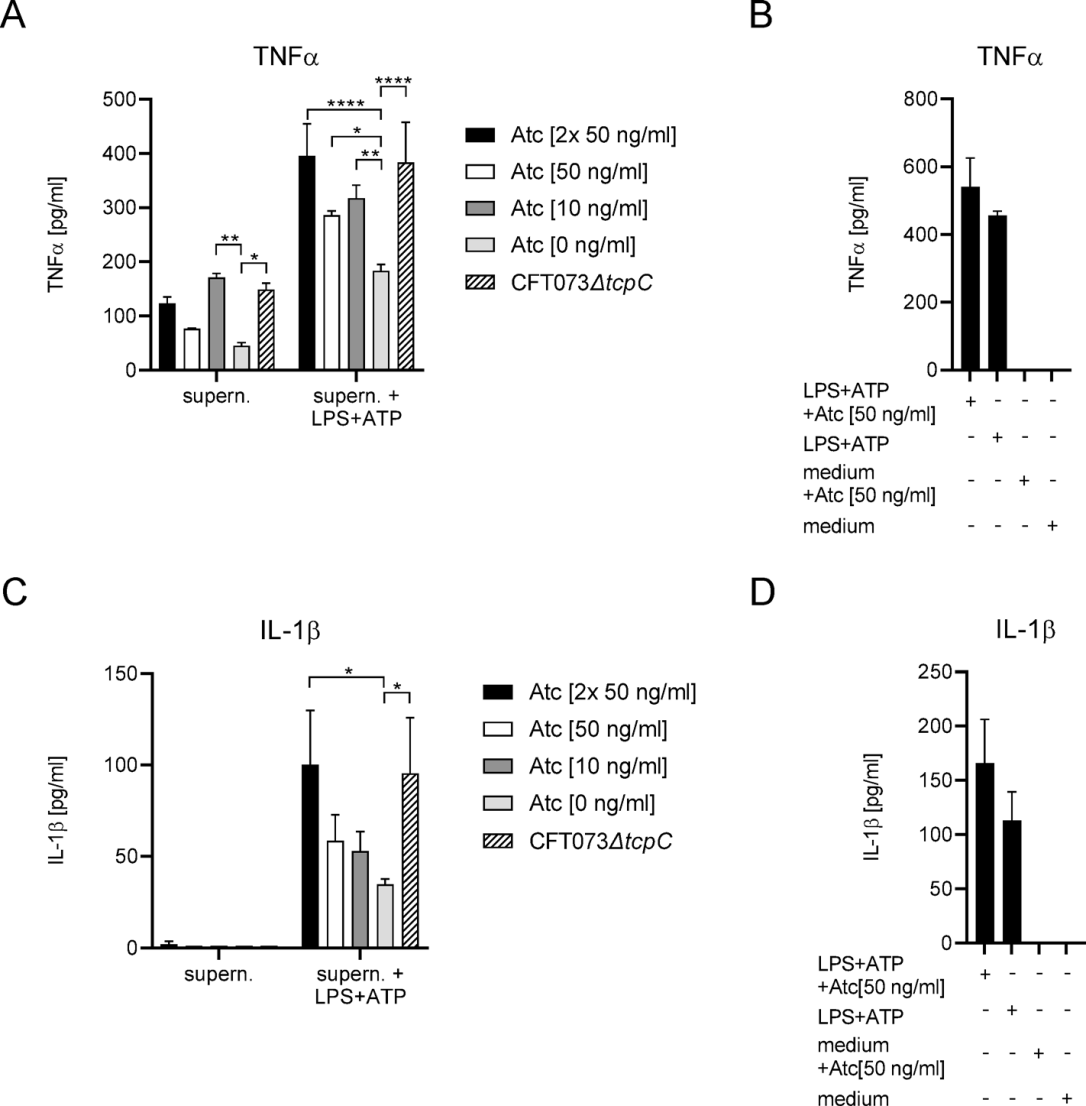
Transfer of CFT073*ΔtcpC* + pASK-IBA5plus-TcpC culture supernatants reduced cytokine secretion by monocytic THP-1 cells. We stimulated monocytic THP-1 cells with culture supernatants of CFT073*ΔtcpC* + pASC-IBA5-TcpC, whose TcpC expression was not or induced with titrated amounts of Atc as indicated. The supernatants were concentrated using a 10 kD cut off filter to remove consumed culture medium and re-diluted with fresh culture medium before they were added to the cell culture. Monocytic THP-1 cells were not or were additionally stimulated with endotoxin plus ATP as indicated. We measured TNFα (**A**,**B**) and IL-1β (**C**,**D**) in culture supernatants. Endotoxin plus ATP +/− Atc served as positive, medium +/- Atc as negative control (**B**,**D**). Graphs depict three replicates. The experiment was repeated with similar results. *P < 0.05, **P < 0.01, ***P < 0.001, ****P < 0.0001, one-way ANOVA, post hoc Tukey.

To circumvent the issue that increasing TcpC amounts influence innate immune signaling but also bacterial replication, we used our inducible bacterial TcpC-expression system, induced the expression of TcpC with ATC or not and transferred the supernatant of CFT073*ΔtcpC* + pASK-IBA5plus-TcpC cultures to monocytic THP-1 cells. In comparison to supernatants from CFT073*ΔtcpC* cultures, supernatants from CFT073*ΔtcpC* + pASK-IBA5plus-TcpC cultures impaired TNFα secretion (Fig. 8A, left part of graph), although TcpC-expression was not induced with Atc. The supernatant presumably contained TcpC since the vector is leaky (Fig. 7A). Induction of TcpC expression with Atc reduced IL-1β suppression, but not in a dose-dependent manner (Fig. 8A, left part of graph). Thus, an Atc concentration of 10 ng/ml did not inhibit TNFα secretion while a concentration of 50 ng/ml did, presumably because of different TcpC/PAMP ratios. To be independent of the transferred PAMPs of the CFT073*ΔtcpC* + pASK-IBA5plus-TcpC cultures, we stimulated monocytic THP-1 cells with endotoxin plus ATP (Fig. 8B, D). Transfer of supernatants from CFT073*ΔtcpC* + pASK-IBA5plus-TcpC cultures in the absence of Atc again demonstrated that TNFα and in this case also IL-1β secretion was impaired (Fig. 8A, C). Increasing doses of Atc reverted this phenotype (Fig. 8A, C). Furthermore, transfer of CFT073 supernatants in comparison to CFT073*ΔtcpC* supernatants significantly impaired the secretion of TNFα by endotoxin plus ATP-stimulated monocytic THP-1 cells (Fig. S3). We also found that the supernatant of CFT073*ΔtcpC* + pASK-IBA5plus-TcpC cultures impaired endotoxin plus ATP-driven cytokine secretion over a wide range of endotoxin concentrations (Fig. S4). We speculate from these results that the TcpC concentration of concentrated supernatants from Atc-uninduced bacterial cultures is relatively high since bacterial replication is not impaired and the reverse situation occurs in Atc-induced bacterial cultures. These relatively high amounts of TcpC are then able to impair innate immune responses by endotoxin plus ATP-stimulated monocytic THP-1 cells.

### The TIR-domain of TcpC impairs cytokine secretion in an FCS-dependent manner

**Fig. 9:**
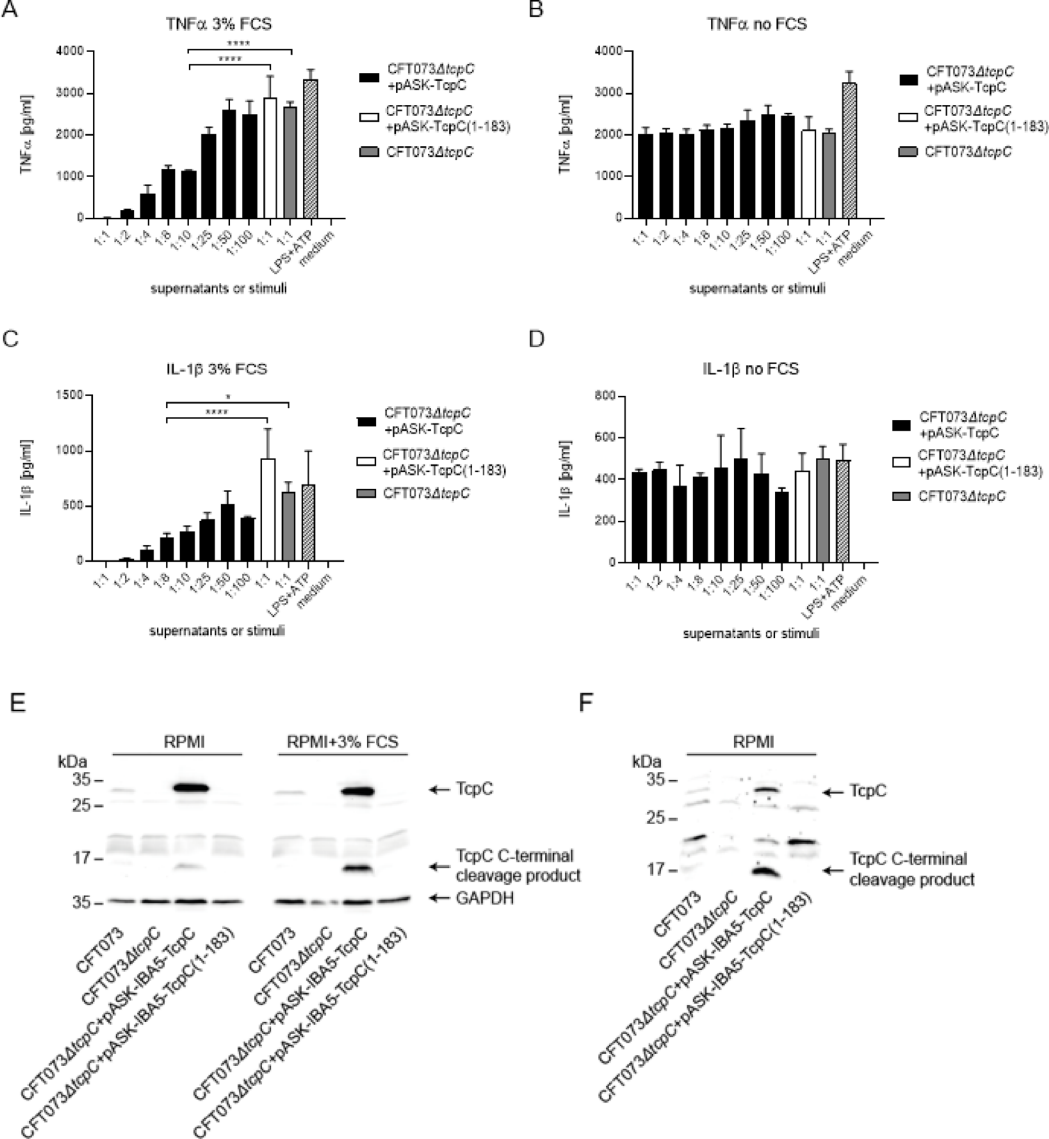
The TIR-domain of TcpC prevents FCS-dependently cytokine secretion by LPS + ATP stimulated monocytic THP-1 cells. We stimulated monocytic THP-1 cells with endotoxin plus ATP in the presence of culture supernatants of CFT073*ΔtcpC* + pASC-IBA5-TcpC (black bars), CFT073*ΔtcpC* + pASC-IBA5-TcpC(1-183) (white bar), or CFT073*ΔtcpC* (gray bar) as indicated. Thus, with the exception of the medium control, THP-1 cells were stimulated with endotoxin plus ATP. We induced TcpC expression in CFT073*ΔtcpC* + pASC-IBA5-TcpC or CFT073*ΔtcpC* + pASC-IBA5-TcpC(1-183) by culture in RPMI in the presence (**A**,**C**) or absence (**B**,**D**) of FCS (3%) for 3 h. Atc was not applied. The supernatants were concentrated using a 10 kD cut off filter for detection of TcpC by Western blot and to remove consumed culture medium. We re-diluted the supernatants with fresh culture medium to the original concentration before they were added to the cell culture. In case of CFT073*ΔtcpC* + pASC-IBA5-TcpC we titrated the supernatant (black bars). We measured TNFα (**A**,**B**) and IL-1β (**C**,**D**) in culture supernatants. Endotoxin plus ATP served as positive (hatched bar), medium as negative control. Graphs depict three replicates. The experiment was repeated twice with similar results. *P < 0.05, **P < 0.01, ***P < 0.001, ****P < 0.0001, one-way ANOVA, post hoc Tukey. Statistically significant differences were also observed for lower than 1:10 or 1:8 dilutions in (**A**) or (C), respectively. We detected TcpC by Western blot in the cytosol of wild type CFT073, or CFT073*ΔtcpC* complemented with either pASC-IBA5-TcpC or pASC-IBA5-TcpC(1-183) (**E**), or in their concentrated culture supernatant (**F**). CFT073*ΔtcpC* served as negative control. Note that the presence or absence of FCS does not change the amount of TcpC in the cytosol. The difference observed in the amount of the C-terminal cleavage product of TcpC was not repeatable. For technical reasons TcpC can only be detected in the bacterial culture supernatant in the absence of FCS, since the concentration of FCS-containing supernatant prevents analysis by Western blot due to protein overload.

To verify that the inhibitory activity of TcpC was dependent on the TIR-domain of the molecule we transformed CFT073*ΔtcpC* with the plasmid pASK-IBA5plus-TcpC(1-183) which encodes only the N-terminal half of TcpC. Transfer of the bacterial culture supernatant to endotoxin plus ATP-stimulated monocytic THP-1 cells failed to inhibit TNFα and IL-1β secretion in contrast to supernatants prepared from CFT073 transformed with the plasmid harboring full length TcpC (Fig. 9A, C). We then tried to detect TcpC in the bacterial culture supernatants and concentrated the supernatant 150-fold using a 10kD cut off filter. However, this step also concentrated the added FCS, which overloaded the Western blot membrane (data not shown). We therefore prepared FCS-free bacterial culture supernatants and tested them first for their inhibitory activity upon transfer to endotoxin plus ATP-stimulated monocytic THP-1 cells. To our surprise, FCS-free supernatants failed to impair cytokine secretion (Fig. 9B, D). However, the cytosol of CFT073 and CFT073*ΔtcpC* + pASK-IBA5plus-TcpC contained the same amount of full length TcpC in the absence or presence of FCS (Fig. 9E). The differences seen for the C-terminal cleavage product of TcpC were not reproducible (Fig. 9E). The culture supernatant of CFT073*ΔtcpC* + pASK-IBA5plus-TcpC prepared in the absence of FCS contained TcpC and the C-terminal cleavage product (Fig. 9F). Whether higher amounts of TcpC are present in bacterial supernatants prepared in the presence of FCS remains to be seen and requires further experiments.

Taken together these experiments demonstrate that the TIR-domain of TcpC impaired cytokine secretion by endotoxin plus ATP-stimulated monocytic THP-1 cells.

## Discussion

Infection of the bladder uroepithelial cell line T24/83 (Figs. 2 and 3), monocytic THP-1 cells and peripheral human blood monocytes (Fig. 4, Fig. S1) with the uropathogenic *E. coli* strain CFT073 elicited significantly higher levels of pro-inflammatory cytokines in comparison to an infection with the *tcpC*-deficient strain CFT073*ΔtcpC*. In contrast, the IL-1β secretion of THP-1 M0 (Fig. 5) and THP-1 M1 macrophages (Fig. 6) were not influenced by TcpC. Interestingly, M2-polarized THP-1 macrophages did not respond to CFT073, although their IL-1β secretion upon stimulation with endotoxin was comparable to M1-polarized THP-1 macrophages, while their TNFα and IL-6 secretion was weaker (Fig. 6). The influence of TcpC on peripheral human blood macrophages was also attenuated considerably (Fig. 5). These findings indicate that in contrast to earlier reports TcpC not only dampens but also stimulates innate immune responses^2–4,7^. Furthermore, its efficacy to modulate cytokine secretion of monocytes varies with their cellular differentiation and polarization. Possibly, differences in the functional status of PBMCs explain why PBMC of one donor are only weakly influenced by TcpC. Also, human epithelial cells of the urinary tract differ in their sensitivity to respond to CFT073: while bladder T24/83 cell release cytokines upon infection, the kidney proximal tube epithelial cell line HK-2 almost not.

### Stimulatory versus inhibitory function of TcpC

To further explore the circumstances, which allow stimulation of innate immune responses, we generated an Atc-inducible TcpC expression vector and transformed it into CFT073*ΔtcpC*. Induction had two consequences: first bacterial expression of TcpC increased but at the same time proliferation of CFT073*ΔtcpC* decreased (Fig. 7A, B). Thus, the ratio of the TcpC output versus bacterial numbers and thereby the amount of PAMPs was changed. Infection of monocytic THP-1 cells with CFT073*ΔtcpC* + pASK-IBA5plus-TcpC, which was induced with increasing Atc concentrations, revealed that IL-1β and to a lower extent TNFα secretion was significantly and dose-dependently augmented (Fig. 7C, D). However, the highest Atc concentration reduced the secretion of the both cytokines. Although monocytic THP-1 cells were infected with the same bacterial burden of CFT073, CFT073*ΔtcpC* or CFT073*ΔtcpC* + pASK-IBA5plus-TcpC (Fig. 7E), Atc attenuated bacterial replication dose-dependently during the infection period thereby presumably reducing the stimulatory potential of the culture (Fig. 7E, F). We speculate that inhibition of replication is caused by the NAD^+^ consuming function of TcpC^17,26^. However, we reported earlier that the induction of the Atc-inducible plasmid pStrep-tag tcpC-E244A transformed into CFT073 still resulted in significant and robust growth retardation of CFT073^27^, although TcpC-TIR E224A was reported to lose its NAD^+^ consuming function^17,28^. Whether these discrepant observations can be explained by the fact that we used a full length TcpC with E244A mutation while the other research groups the TIR-domain of TcpC with E244A mutation remains an open question.

The inhibitory function of TcpC was revealed in transfer experiments. Thus, transfer of CFT073*ΔtcpC* + pASK-IBA5plus-TcpC culture supernatants to monocytic THP-1 cells in the absence or presence of an additional endotoxin plus ATP stimulation reduced TNFα secretion in comparison to supernatants of CFT073*ΔtcpC* cultures (Fig. 8A). To detect the inhibitory influence of TcpC on IL-1β secretion, stimulation of monocytic THP-1 cells with endotoxin and ATP was required, as expected (Fig. 8C). We observed the highest suppression in case the plasmid pASK-IBA5-TcpC was not induced by Atc, thus, only the amount of TcpC under leaky conditions was present. Possibly this condition produced the highest amount of secreted TcpC, since bacterial growth was not impaired. Transfer experiments also revealed that endogenous TcpC expression levels sufficed for inhibition as CFT073 culture supernatants inhibited TNFα-secretion significantly in comparison to CFT073*ΔtcpC* culture supernatants (Fig. S3).

The inhibitory impact of TcpC was dependent on its TIR-domain (Fig. 9). This result was expected as we described earlier that the recombinant TIR-domain of TcpC was able to impair TNFα-secretion induced by TLR-ligands such as endotoxin, Pam3Cys, flagellin and CpG-DNA^2^. However, the inhibitory effect of transferred TcpC depended on the presence of FCS during the expression of the protein. The influence of FCS on TcpC function is unclear. While cytosolic production of TcpC was not affected by FCS (Fig. 9), the amount of TcpC in the culture supernatant may differ. Further immune precipitation experiments will address this issue.

Taken together TcpC is stimulatory in the context of an infection of monocytes with CFT073 while it is inhibitory upon transfer of culture supernatants to monocytes.

### TcpC modulates the TLR4-dependent pathogen recognition pathway

We reported previously that TcpC impairs TNF-secretion by murine bone marrow-derived macrophages MyD88-dependently^2^. In vivo, urinary tract infection with CFT073 increased urinary levels of macrophage inflammatory protein 2 (MIP-2) in a MyD88- and TLR4-dependent manner^7^. The effect of TcpC on MIP-2 was also MyD88- and TLR4-dependent, indicating that the TLR4-signaling cascade is a critical checkpoint manipulated by TcpC. Using the human bladder epithelial cell line T24/83 we show that the CFT073-induced secretion of IL-6 and TNFα depended on MyD88 but the impact of TcpC was still detectable (Fig. 2). It thus appears that the relevance of MyD88 for the function of TcpC to modulate TLR4-signaling is cell type dependent.

### TcpC checkpoint modulation depends on monocyte differentiation and polarization

Another new finding of this study is the observation that the effect of TcpC on innate immune responses by monocytes is reduced upon their cellular differentiation. Thus, differentiation of monocytic THP-1 cells or peripheral blood monocytes to macrophages completely impaired or reduced their sensitivity for TcpC, respectively (Figs. 4 and 5). Possibly, this phenomenon might be explained by the much higher endotoxin sensitivity of macrophages compared to monocytes (Figs. 4 and 5), which was already observed previously by Seow et al.^29^. This group also reported that the complement component C5a amplified the TLR4-mediated endotoxin-response of monocytes but suppressed the one of macrophages^29^, indicating that the TLR4 signaling chain can be influenced in a cell differentiation-dependent manner. Pan et al. reported recently that TcpC promotes the polarization of kidney macrophages to the M2 but impairs the M1 phenotype during a urinary tract infection with CFT073 or CFT073*ΔtcpC*^30^. They also showed that recombinant TcpC impaired TNFα secretion by M1- and to a lower extent by M2-polarized THP-1 macrophages but promoted IL-10 secretion by M2-polarized THP-1 macrophages. In their experiments, recombinant TcpC was added during the polarization phase. In contrast, we explored the influence of TcpC on already polarized macrophages. Polarized M1 THP-1 macrophages were resistant to TcpC’s influence on IL-1β but not TNFα or IL-6 secretion during an infection with CFT073. The TNFα response was not significantly changed by TcpC, but a trend was visible. Surprisingly, M2 polarized THP-1 cells did not react to CFT073 although they responded to endotoxin derived from *E. coli*. The reason for this phenomenon is presently unclear but it shows that stimulation of cells with CFT073 or endotoxin differs.

In conclusion, differentiation and polarization of monocytes to macrophages severely influence their sensitivity to TcpC. Moreover, the effect of TcpC on innate immunity differs during an infection versus an endotoxin-mediated stimulation of innate immune cells. In the first condition, innate immune responses are strengthened, in the latter impaired.

## Supplementary Information

Below is the link to the electronic supplementary material.

Supplementary Material 1

Supplementary Material 2

Supplementary Material 3

## Data Availability

Data are available on request from the authors. Please contact LH or TM.
