# Supplementary material for: TIR-domain-containing protein C as modulator of innate immune checkpoints
Source: Sci Rep. 2025 Nov 27;15:42562. doi: 10.1038/s41598-025-29677-w (PMC12663425; doi:10.1038/s41598-025-29677-w)
Supplement: Supplementary file 1 — Supplementary Material 1 [file 41598_2025_29677_MOESM1_ESM.docx]

**Figure S1.** Growth inhibition of CFT073 by induced TcpC expression depended substantially on the TIR-domain of TcpC. Growth of CFT073*ΔtcpC*+pASK-TcpC or CFT073*ΔtcpC*+pASK-TcpC(1-183) induced with Atc (50 ng/ml) or not, respectively. We cultured bacteria in LB medium and recorded OD_600_ values every hour.

**Figure S2.** (A) Induced and endogenous expression of TcpC suppresses significantly TNFα secretion by monocytic THP-1 cells upon stimulation with endotoxin+ATP in the presence or absence of CFT073 culture supernatants as indicated. The bacterial culture supernatants were filtered through a 10 kD cut off filter to remove consumed culture medium. Supernatants were then diluted with fresh culture medium and added to the stimulated monocytic THP-1 cells. We also determined bacterial replication before (B) and after (C) induction of pASK-TcpC with Atc.

* *P*<0.05, **** <0.0001, ANOVA, post hoc Tukey.

**Figure S3.** CFT073*ΔtcpC*+pASK-TcpC-conditioned medium impaired LPS+ATP-mediated TNFα (A) and IL-1β secretion (B). We stimulated monocytic THP-1 cells with different concentrations of LPS and added ATP in the presence or absence of CFT073*ΔtcpC*+pASK-TcpC-conditioned (Atc 0 ng/ml) medium as indicated in the graphs. The bacterial culture supernatants were concentrated using a 10 kD cut off filter to remove consumed culture medium, rediluted with fresh culture medium and then used to stimulate monocytic THP-1 cells.
