# Supplementary material for: TIR-domain-containing protein C as modulator of innate immune checkpoints
Source: Sci Rep. 2025 Nov 27;15:42562. doi: 10.1038/s41598-025-29677-w (PMC12663425; doi:10.1038/s41598-025-29677-w)

Figure 7A exposure time 10s

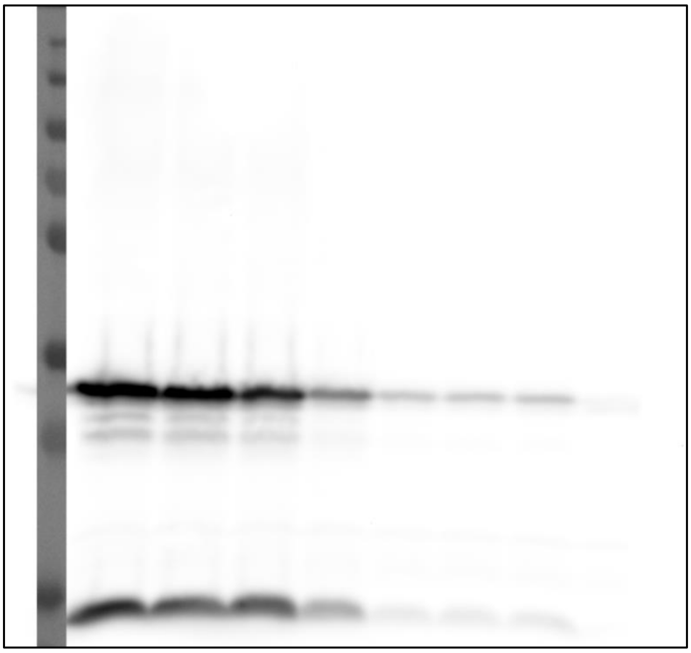

Figure 7A markers

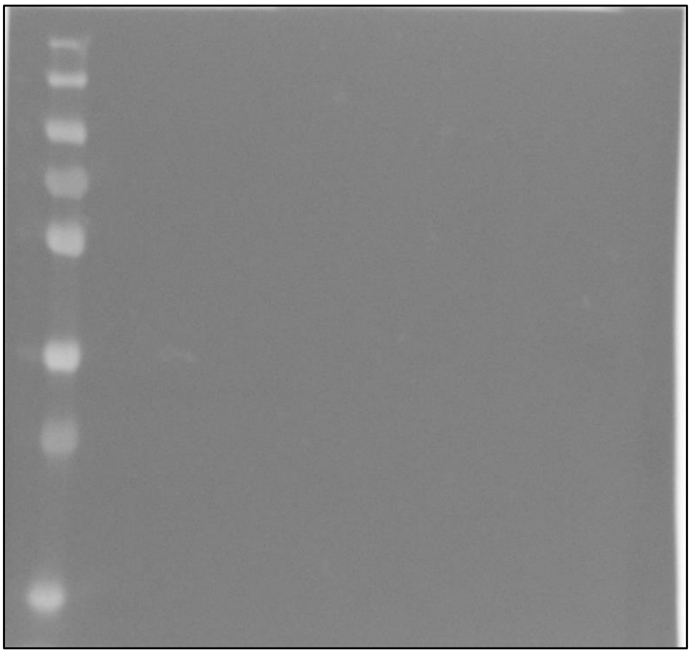

Figure 9E GAPDH exposure time 30s

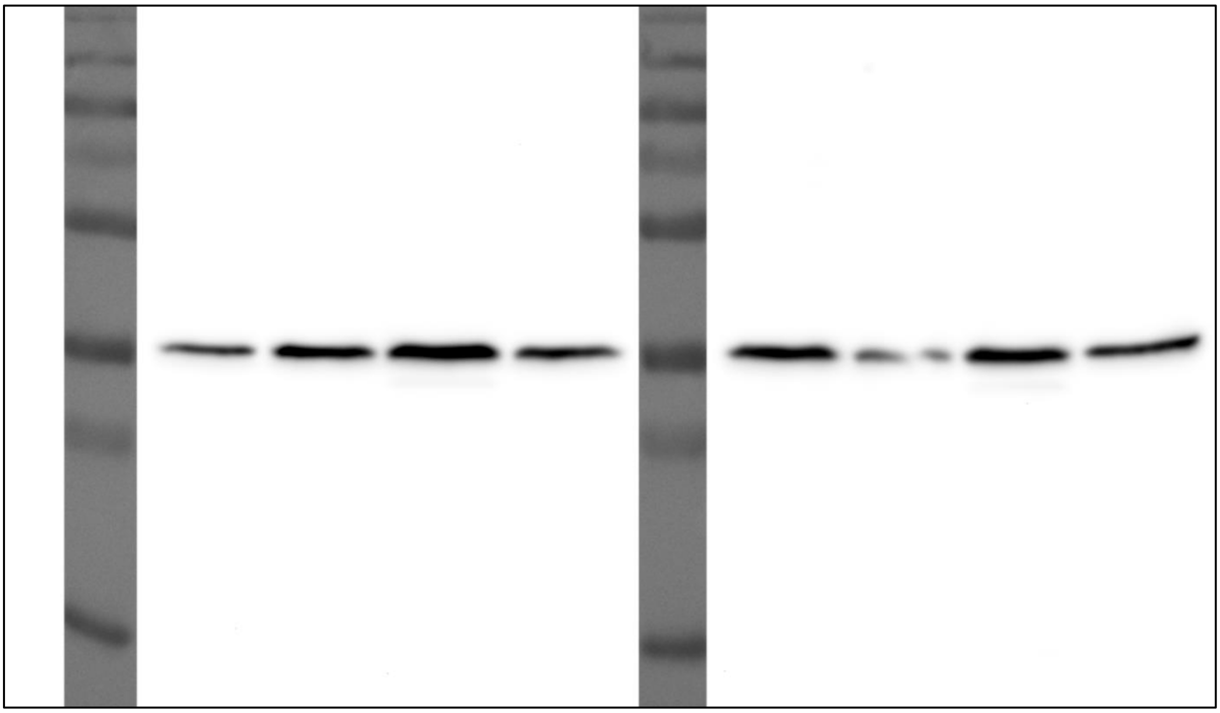

Figure 9E GAPDH markers

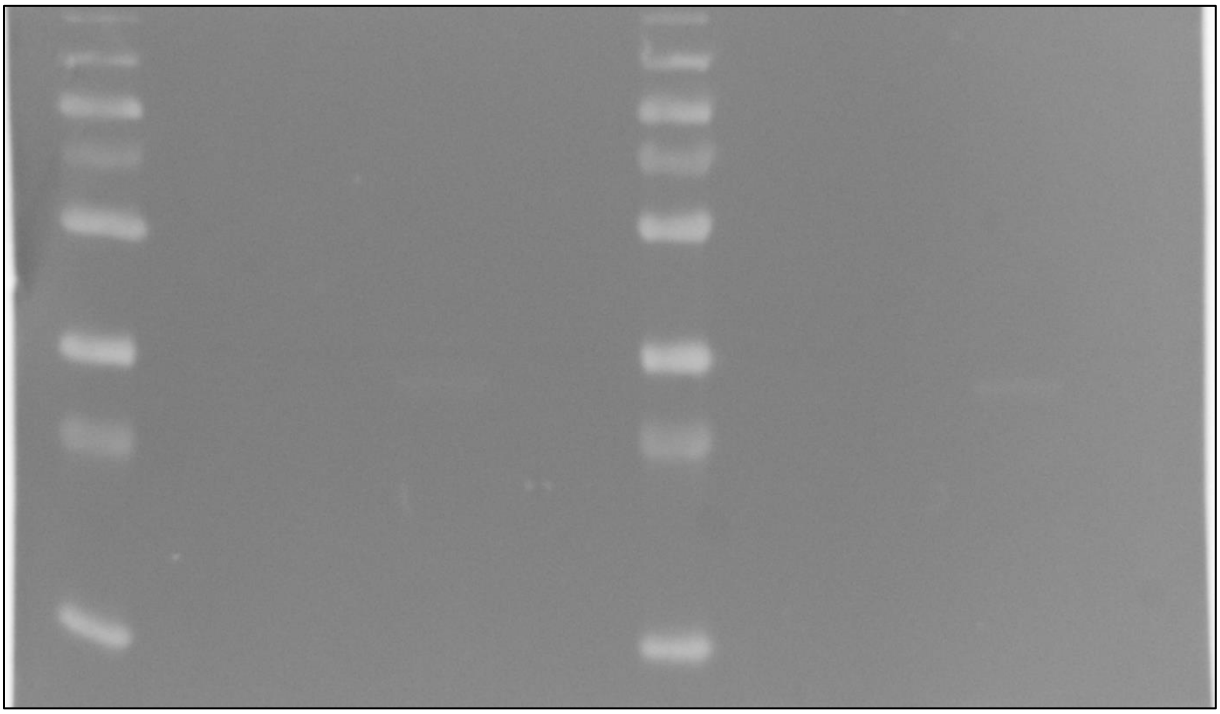

Figure 9E TcpC exposure time 5min

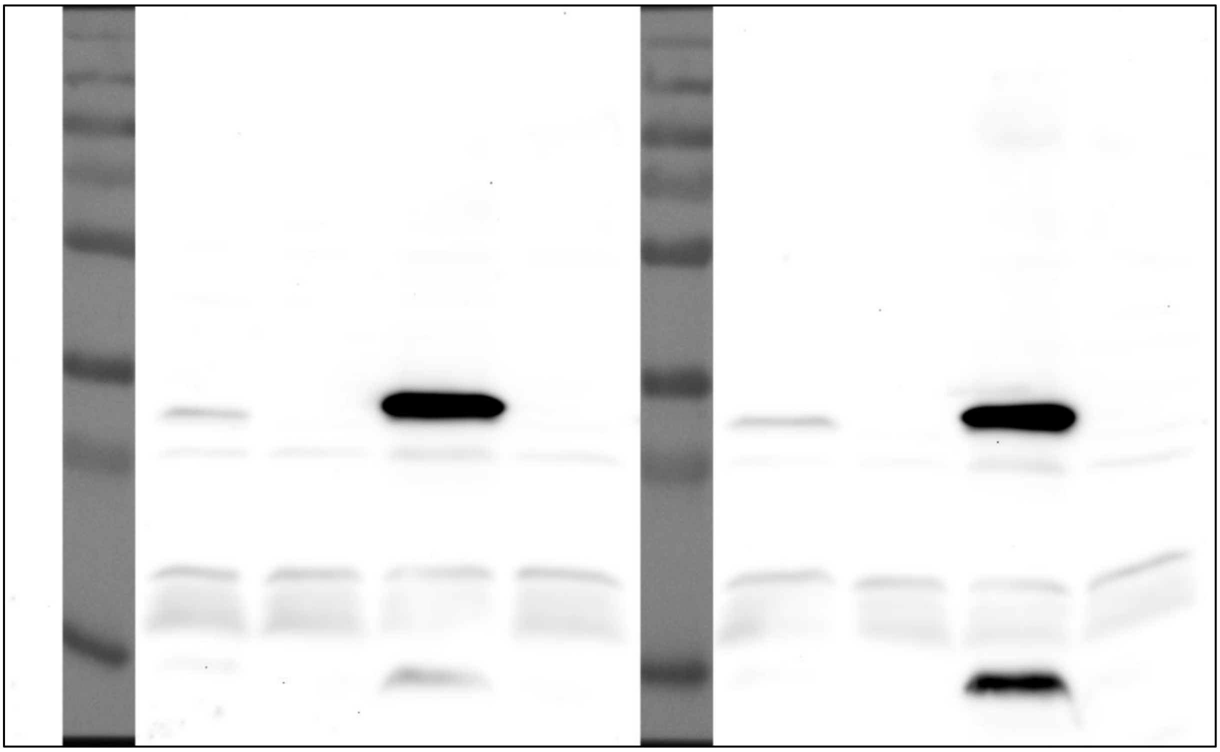

Figure 9E TcpC marker

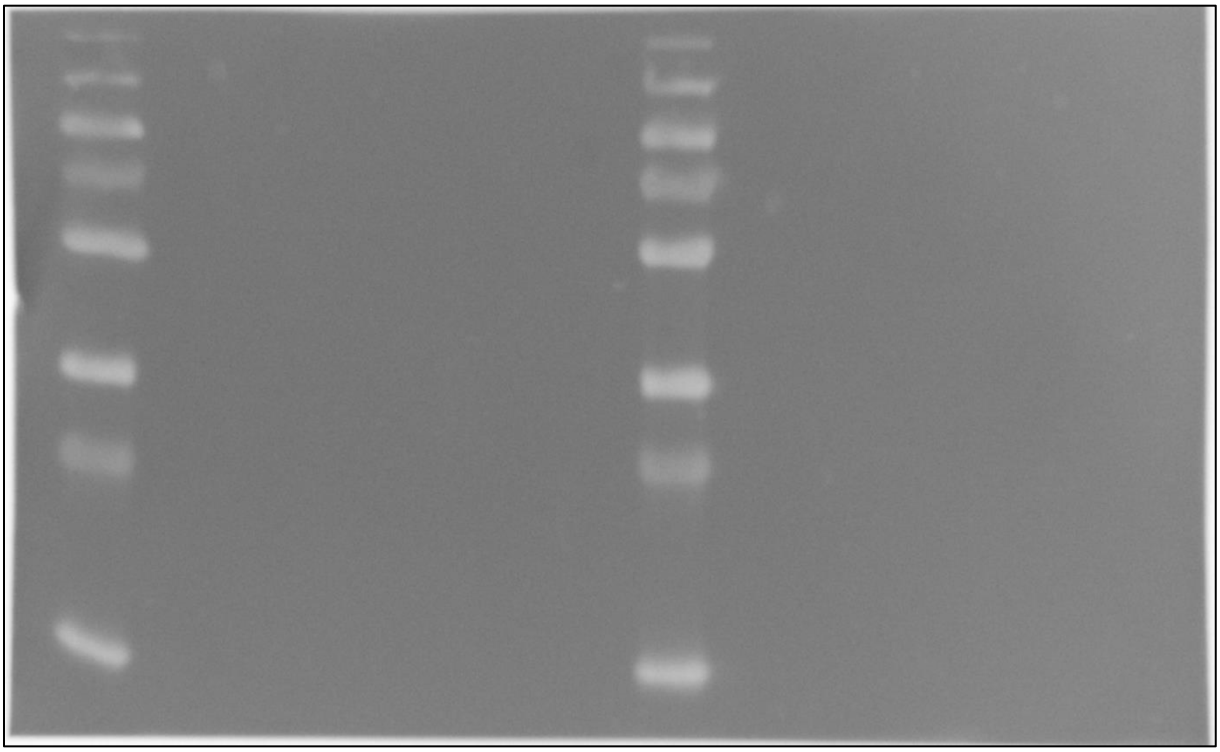

Figure 9F exposure time 20min

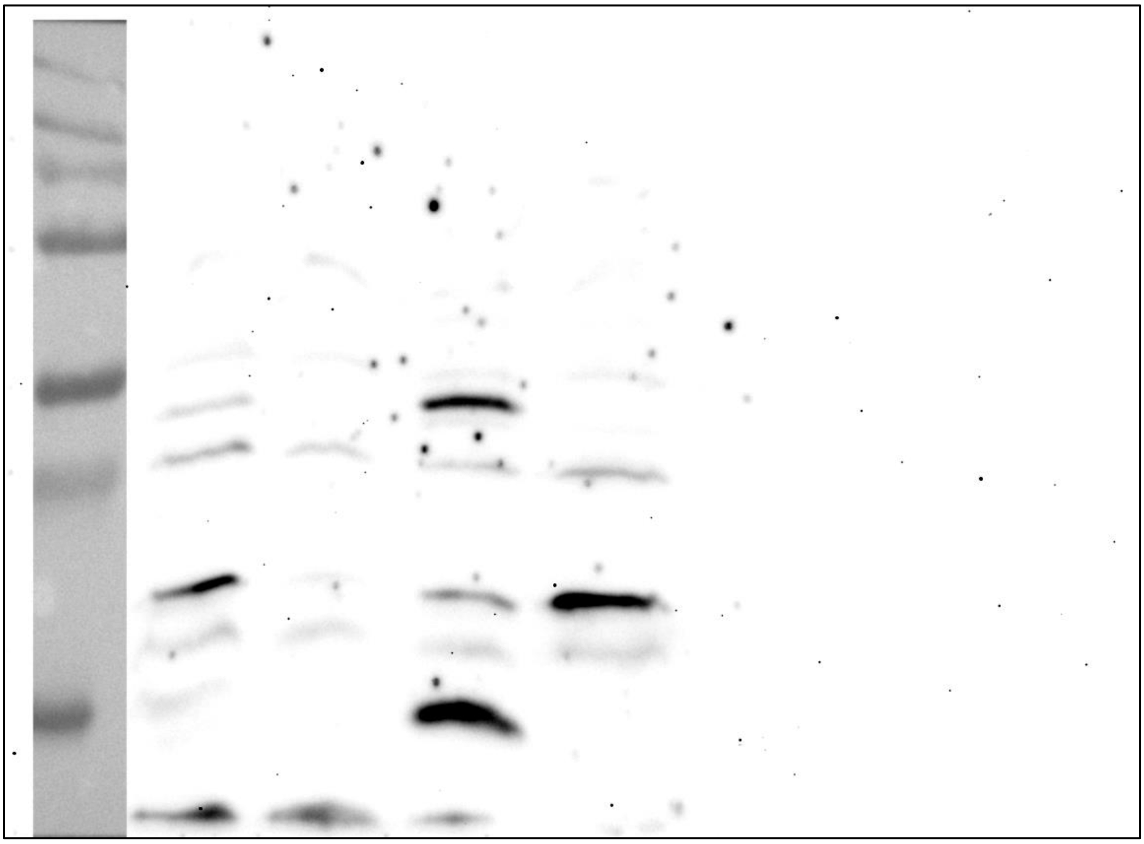

Figure 9F markers

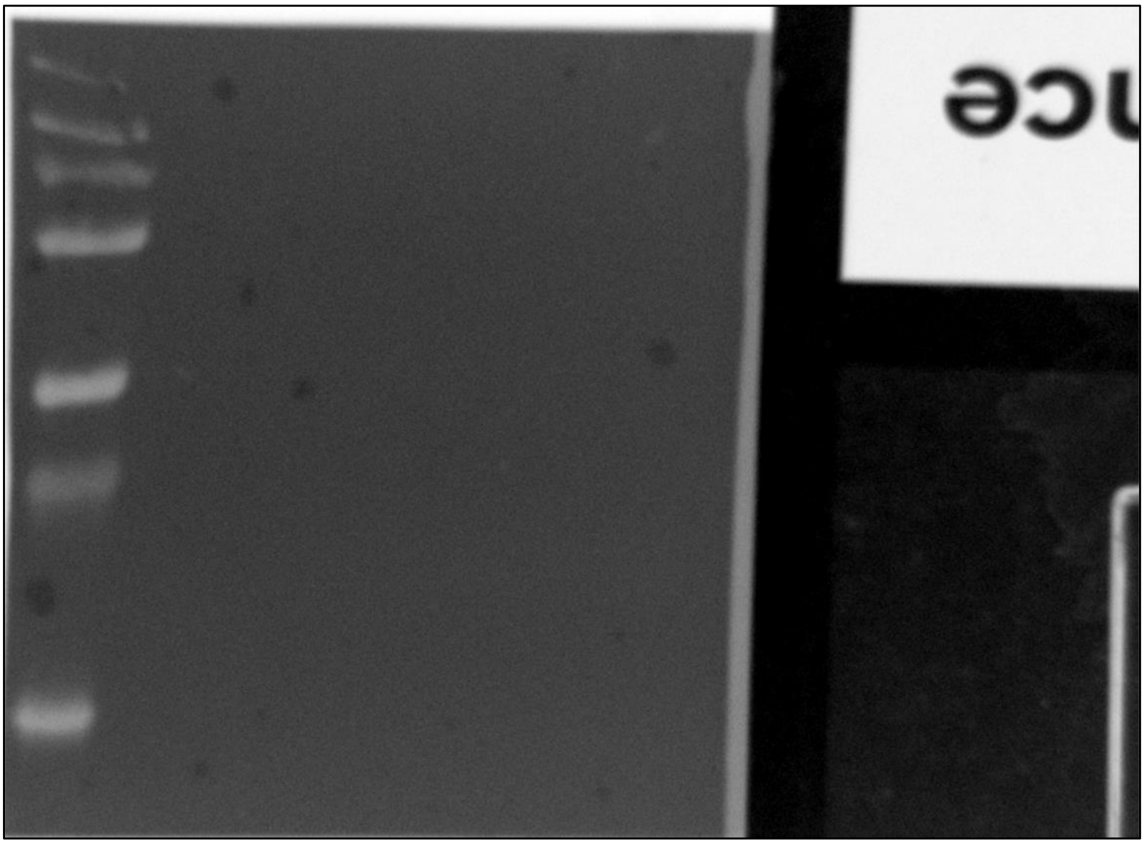

Supplement: Supplementary file 2 — Supplementary Material 2 [file 41598_2025_29677_MOESM2_ESM.pdf]
