# Supplementary material for: TIR-domain-containing protein C as modulator of innate immune checkpoints
Source: Sci Rep. 2025 Nov 27;15:42562. doi: 10.1038/s41598-025-29677-w (PMC12663425; doi:10.1038/s41598-025-29677-w)
Supplement: Supplementary file 3 — Supplementary Material 3 [file 41598_2025_29677_MOESM3_ESM.docx]

**Figure S1.** TcpC harnesses CFT073 induced immune responses by PBMC and peripheral blood monocytes. Peripheral blood mononuclear cells from three healthy blood donors were infected for 5h with CFT073, CFT073*ΔtcpC* or CFT073*ΔtcpC*+pTcpC with different MOIs as indicated. We determined IL-1β (A-C) and TNFα (D-F) in the culture supernatant. Graphs in (A-F) depict three replicates. Peripheral blood monocytes from the same healthy blood donors were infected as described for PBMCs and IL-1β (G-I) and TNFα levels (J-L) of culture supernatants were determined. Graphs in (G-L) depict three replicates. LPS+ATP served as positive, medium as negative control. **P*<0.05, ***P*<0.01, ****P*<0.001, *****P*<0.0001, two-way ANOVA, post hoc Tukey.

**Figure S2.** Growth inhibition of CFT073 by induced TcpC expression depended substantially on the TIR-domain of TcpC. Growth of CFT073*ΔtcpC*+pASK-TcpC or CFT073*ΔtcpC*+pASK-TcpC(1-183) induced with Atc (50 ng/ml) or not, respectively. We cultured bacteria in LB medium and recorded OD_600_ values every hour.

**Figure S3.** (A) Induced and endogenous expression of TcpC suppresses significantly TNFα secretion by monocytic THP-1 cells upon stimulation with endotoxin+ATP and in the presence or absence of transferred CFT073 culture supernatants as indicated. The bacterial culture supernatants were filtered through a 10 kD cut off filter to remove consumed culture medium. Supernatants were then diluted with fresh culture medium and added to the stimulated monocytic THP-1 cells. We also determined bacterial replication before (B) and after (C) induction of pASK-TcpC with Atc.

* *P*<0.05, **** <0.0001, ANOVA, post hoc Tukey.

**Figure S4.** CFT073*ΔtcpC*+pASK-TcpC-conditioned medium impaired LPS+ATP-mediated TNFα (A) and IL-1β secretion (B). We stimulated monocytic THP-1 cells with different concentrations of LPS and added ATP in the presence or absence of CFT073*ΔtcpC*+pASK-TcpC-conditioned (Atc 0 ng/ml) medium as indicated in the graphs. The bacterial culture supernatants were concentrated using a 10 kD cut off filter to remove consumed culture medium, rediluted with fresh culture medium and then used to stimulate monocytic THP-1 cells.
